# The effect of control measures on COVID-19 transmission in Italy: Comparison with Guangdong province in China

**DOI:** 10.1186/s40249-020-00730-2

**Published:** 2020-09-16

**Authors:** Pei-Yu Liu, Sha He, Li-Bin Rong, San-Yi Tang

**Affiliations:** 1 School of Mathematics and Information Science, Shaanxi Normal University, Xi’an, 710119 PR China; 2 Department of Mathematics, University of Florida, Gainesville, 32601 USA

**Keywords:** COVID-19, Cumulative cases, Control measures, Model prediction

## Abstract

**Background:**

COVID-19 has spread all around the world. Italy is one of the worst affected countries in Europe. Although there is a trend of relief, the epidemic situation hasn’t stabilized yet. This study aims to investigate the dynamics of the disease spread in Italy and provide some suggestions on containing the epidemic.

**Methods:**

We compared Italy’s status at the outbreak stage and control measures with Guangdong Province in China by data observation and analysis. A modified autonomous SEIR model was used to study the COVID-19 epidemic and transmission potential during the early stage of the outbreak in Italy. We also utilized a time-dependent dynamic model to study the future disease dynamics in Italy. The impact of various non-pharmaceutical control measures on epidemic was investigated through uncertainty and sensitivity analyses.

**Results:**

The comparison of specific measures implemented in the two places and the time when the measures were initiated shows that the initial prevention and control actions in Italy were not sufficiently timely and effective. We estimated parameter values based on available cumulative data and calculated the basic reproduction number to be 4.32 before the national lockdown in Italy. Based on the estimated parameter values, we performed numerical simulations to predict the epidemic trend and evaluate the impact of contact limitation, detection and diagnosis, and individual behavior change due to media coverage on the epidemic.

**Conclusions:**

Italy was in a severe epidemic status and the control measures were not sufficiently timely and effective in the beginning. Non-pharmaceutical interventions, including contact restrictions and improvement of case recognition, play an important role in containing the COVID-19 epidemic. The effect of individual behavior changes due to media update of the outbreak cannot be ignored. For policy-makers, early and strict blockade measures, fast detection and improving media publicity are key to containing the epidemic.

## Background

**Fig. 1:**
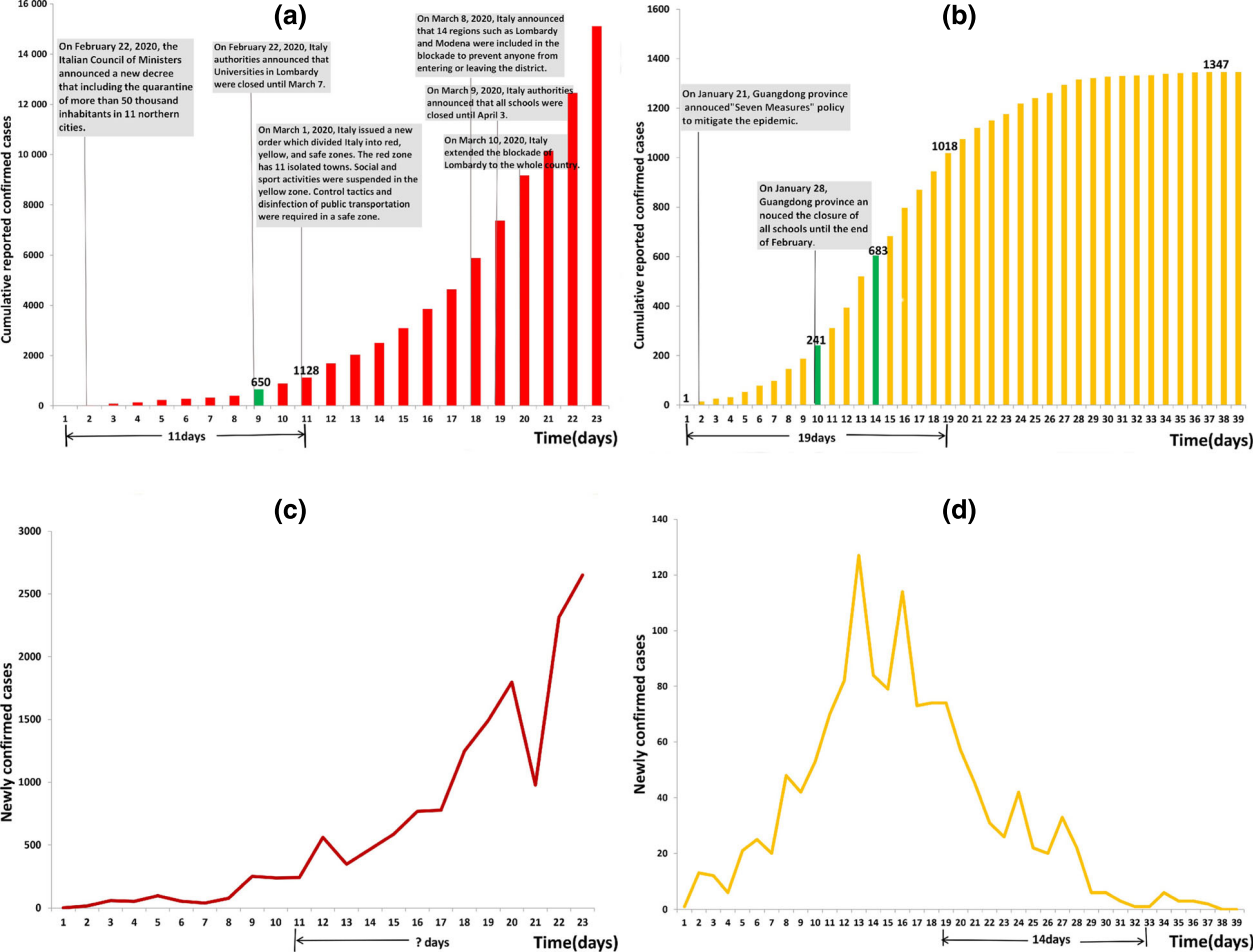
Cumulative reported cases, control measures and newly confirmed cases in Italy and Guangdong. (**a**) The cumulative reported cases and time-line of the changes of control strategy in Italy. (**b**) The cumulative reported cases and time-line of the changes of control strategy in Guangdong. (**c**) Newly confirmed cases in Italy. (**d**) Newly confirmed cases in Guangdong. The x-coordinate represents time (days) during the period from Feb 20 to Mar 13 in Italy and Jan 19 to Feb 26 in Guangdong

Six types of coronavirus have been found to be capable of causing human infections [1]. Four of them are not highly pathogenic, typically causing cold symptoms in immunocompetent individuals, while the other two types, the severe acute respiratory syndrome (SARS) and the Middle East respiratory syndrome (MERS), can result in severe respiratory illness and fatalities [2–4]. In late 2019, a novel coronavirus COVID-19, which turns out to be more infectious and can survive higher temperature than SARS [5], has been identified as the pathogen of an ongoing pandemic. This virus has spread to many countries in the world [6–9]. By May 31, 2020, more than 489 921 confirmed cases due to COVID-19 had been reported in Eastern Mediterranean [10], Italy is one of the most hit countries with more than 232 664 confirmed cases, which account for 47% of reported cases in Eastern Mediterranean. Although there is a trend of relief in Italy but the situation remains unstable. Control measures have been implemented in Italy aiming to contain the outbreak and the time-line of the changes of control strategy in Italy is shown in Fig. 1a.

In the course of dealing with the worldwide outbreak crisis, mathematical models have played an important role in providing policymakers with timely and crucial epidemiological information. Many models have been developed, for instance, using the transmission model of SARS [11, 12] to investigate the spread of SARS, the molecular SARS model to understand its molecular structure for biochemical function and drug design [13], and animal models of MERS to study how disease passes from animals to humans [14, 15]. The media impact models illustrate how social media influences the propagation of SARS [16]. Mathematical models have also been developed to study the transmission of COVID-19 in response to this current crisis [17–20]. For example, He et al. have developed a discrete-time stochastic epidemic model with binomial distributions to study the transmission of COVID-19 [21]. Tang et al. used a deterministic compartmental model to investigate the COVID-19 epidemic in the mainland of China, the Guangdong province of China, and Republic of Korea [22, 23]. The control measures and the timings of initiation in China might provide some suggestions to other countries [24]. Fang et al. adopted the model of susceptible-exposed-infectious-recovered (SEIR) with a data-driven analysis to study the effectiveness of government interventions in China [25].

What’s the transmission dynamics of the COVID-19 and what is the impact of non-pharmaceutical control measures on the COVID-19 epidemic in Italy? In order to address the above problems in more detail, we choose Guangdong Province (the most developed province in China, and the population is nearly twice that of Italy [26, 27]) for comparative study to reveal the timeliness and effectiveness of the comprehensive prevention and control strategies. In this paper, we compare the strategies in the two places by analyzing their specific control measures, the change of cumulative cases, new cases, prevalence ratio and cure ratio, and further reveal the epidemic situation in the world and Europe through the observation and analysis of cumulative confirmed cases. We develop a modified autonomous SEIR model to investigate the early transmission dynamic in Italy. Considering the continuous effects of prevention actions, we incorporate piecewise functions for the contact and diagnosis rate into previous autonomous model and develop a time-dependent dynamic model. We assess the impact of the control measure’s delayed effect, the decrease rate in the contact rate, the diagnosis rate under different control intensity and the strength of the public’s awareness by uncertainty and sensitivity analyses. We also predict the peak time and number of cases under various measures. At last, based on parameter estimates we evaluate the prediction by fitting the model to the latest data up to May 31, 2020.

## Methods

### Collection of cases data

We obtained data of confirmed COVID-19 cases that were reported in Guangdong province, Italy and other places in the world from the Health Commission of Guangdong Province, the Ministry of Health of Italy, and the World Health Organization [28–31]. The data include infected cases in the world and Europe since January 26 to March 12, 2020, the cumulative confirmed cases, newly reported cases, death cases, and recovered cases in Italy from January 30 to March 13, 2020 and in Guangdong from January 19 to February 26, 2020. The number of cumulative confirmed cases in Italy was 2 between January 30 and February 5, 2020, and was 3 from February 5 to February 19, 2020. The numbers of cumulative death and recovered cases were 0 from January 30 to February 19, 2020. We exclude the data in these periods and will focus on the Italian data between February 20 and March 13 because this is the early stage of the Italian outbreak, which allows us to compare the effectiveness of the prevention and control measures used in Italy and Guangdong by analysis of those datasets.

### Time-line of control measures

In Fig. 1, we select February 20 as the first day when there were 4 cumulative confirmed cases in Italy, and January 19 as the first day when Guangdong had one cumulative confirmed case. On February 28 Italy closed schools in Lombardy, the most serious epidemic area, when the number of cumulative confirmed cases was already 650 (Fig. 1a). This number is similar to the confirmed cases (i.e. 683) in Guangdong on February 2. In comparison, the Guangdong government took “Seven Measures” policy (e.g. blockade of unnecessary public places) on the third day (January 21) to prevent the spread of the epidemic and shut down all schools on January 28, when there were only 241 cumulative confirmed cases (Fig. 1b). Therefore, Italy closed schools about 5 days (January 28 − February 2) later than Guangdong. In Fig. 1c we see that the number of cumulative cases has already exceeded 10 thousand in Italy, however, the newly confirmed cases in Guangdong began to decline since the 37th day and reached the peak of cumulative cases at only 1347 (Fig. 1d). In summary, it took 14 days for Guangdong’s reported cases to decline since its cumulative confirmed cases exceeded 1000.

### Model

In the early stage of the epidemic in Italy, the government did not take many prevention measures across the country. Thus, to study the epidemic of this stage, we extend the classical deterministic susceptible-exposed-infectious-removed (SEIR) epidemic model by dividing the population into susceptible (*S*), exposed (*E*), symptomatic/asymptomatic infected (*I*/*A*), confirmed (*H*) and recovered (*R*) compartments. The susceptible and exposed populations are further partitioned into quarantined susceptible (*S*_*q*_) and quarantined suspected individuals (*E*_*q*_). Based on the previous research [22], we adopt an autonomous model to study the early stage of the outbreak. We assume that the individuals exposed to the virus are quarantined with a proportion *q* by contact tracing. If the quarantined individuals are successfully infected, they will move to *E*_*q*_ compartment, otherwise they move to the *S*_*q*_ compartment. The individuals who exposed to the virus but were missed in the contact tracing with rate 1−*q* can either move to the compartment *E* or still stay in compartment *S*, depending on whether they are infected or not. We assume that the successful transmission probability is *β* and the contact rate is c. The infected individuals can be detected and then isolated at a rate of *δ*_*I*_ or move to the compartment *R* at the rate of *γ*_*I*_ due to recovery. The death rate of the infectious individuals with symptoms *I* and the isolated infected individuals *H* is *α*. We also assume that the asymptomatic infectious is neither dead nor hospitalized. With these assumptions the model can be described by

1 $$ \begin{array}{l} \frac{dS}{dt}=-\left(\beta c+cq(1-\beta)\right)S\frac{(I+\theta A)}{N}+\lambda S_{q},\\ \frac{dE}{dt}=\beta c (1-q)S\frac{(I+\theta A)}{N}-\sigma E,\\ \frac{dI}{dt}=\sigma \rho E-\left(\delta_{I}+\alpha+\gamma_{I}\right)I,\\ \frac{dA}{dt}=\sigma(1-\rho)E-\gamma_{A}A,\\ \frac{{dS}_{q}}{dt}=(1-\beta)cqS\frac{(I+\theta A)}{N}-\lambda S_{q},\\ \frac{{dE}_{q}}{dt}=\beta cqS\frac{(I+\theta A)}{N}-\delta_{q}E_{q},\\ \frac{dH}{dt}=\delta_{I}I +\delta_{q}E_{q}-\left(\alpha+\gamma_{H}\right)H,\\ \frac{dR}{dt}=\gamma_{I}I+\gamma_{A}A+\gamma_{H}H.\\ \end{array}  $$

The more detailed definitions of variables and parameters for model (1) are provided in Table 1. As the population size is much larger than the size of the outbreak, i.e. *S*(*t*)/*N*≈1, the basic reproductive number *R*_0_ of model (1) is given by the following formula by utilizing the next generation matrix [32].

$$R_{0}=\frac{\beta\rho c(1-q)}{\delta_{I}+\alpha+\gamma_{I}}+\frac{\beta(1-\rho) c\theta(1-q)}{\gamma_{A}}. $$

**Table 1:** Estimates of parameters and initial values of variables in model (1)

Parameters	Definition	value	Source
*c*	Initial contact rate	15	Estimated
*β*	Probability of successful transmission	0.1334	Estimated
*θ*	Transmission probability reduction of asymptomatically infected individuals	0.1	Estimated
*ρ*	Ratio of symptomatic infection	0.6	Estimated
*q*	Quarantine rate of exposed individuals	0.415	Estimated
*σ*	Transition rate of exposed individuals to the infected class	1/7	[33]
*λ*	Rate at which the quarantined uninfected contacts are released	1/14	[22]
*δ*_*I*_	Transition rate of symptomatically infected individuals to the quarantined infected class	0.2257	Estimated
*δ*_*q*_	Transition rate of quarantined exposed individuals to the quarantined infected class	0.2	Estimated
*γ*_*I*_	Recovery rate of symptomatically infected individuals	0.02	Estimated
*γ*_*A*_	Recovery rate of asymptomatically infected individuals	0.07	Estimated
*γ*_*H*_	Recovery rate of quarantined infected individuals	0.0239	Estimated
*α*	Disease-induced death rate	0.013	Estimated
*N*	The total population	6.048×10^7^	Data
*E*(0)	Initial exposed population	26	Estimated
*I*(0)	Initial symptomatically infected population	20	Estimated
*A*(0)	Initial asymptomatically infected population	5	Estimated
*S*_*q*_(0)	Initial quarantined susceptible population	51	Estimated
*E*_*q*_(0)	Initial quarantined exposed population	13	Estimated
*H*(0)	Initial quarantined infected population	3	Data
*R*(0)	Initial recovered population	0	Data

#### Time-dependent model

The above model will be used to study the early stage of the outbreak. However, with a series of prevention and control measures being implemented by the government, the autonomous model needs to be modified. Because of the difference before and after the implementation of control measures, piecewise functions of the contact rate and diagnosis rate are introduced to the autonomous model.

The contact rate is a constant in the autonomous model, i.e. the average number of susceptible individuals that an exposed people can contact without any control measures in a unit time. As the action of regional or national lockdown came into effect, people’s contact will gradually decrease. Thus, we assume that the contact rate is an exponential decreasing function of time *t* after the government has taken the control measures. The contact rate *c*(*t*) is assumed to take the following form:

2 $$ c(t)=\left\{\begin{array}{l} c_{0}, \ \ t\leq t^{*}+\tau,\\ \left(c_{0}-c_{b}\right)e^{-r_{1}(t-t^{*}-\tau)}+c_{b}, \ \ t>t^{*}+\tau.\\ \end{array}\right.  $$

Here *c*_0_ denotes the contact rate at the initial time without control measures, *c*_*b*_ denotes the minimum contact rate under the current control strategies (*c*_*b*_<*c*_0_). Clearly, *c*(0)=*c*_0_, $\mathop {lim}\limits _{t\rightarrow \infty }{c(t)}=c_{b}$. The parameter *r*_1_ in the exponential decreasing rate *c*(*t*) measures how fast the contact rate decreases under control measures. The data we used for fitting started from February 20, 2020 and Italy was blockaded on March 10, 2020. Thus, we let *t*^∗^=18. Since the control measures cannot come into effect immediately after the implementation, we add a time lag *τ* which represents the delayed effect of prevention actions (the earlier and stricter the implementation actions, the smaller the *τ*).

Similarly, because the efficiency of detection and availability of medical resources vary, we assume that the diagnosis rate is a time-dependent piecewise function rather than a constant. It is an increasing function when medical resources are adequate and a decrease function when they are not. The duration of diagnosis 1/*δ*_*I*_(*t*) is given by the following form:

3 $$ \frac{1}{\delta_{I}(t)}=\left\{\begin{array}{l} \frac{1}{\delta_{I0}}, \ \ t\leq t^{*},\\ \left(\frac{1}{\delta_{I0}}-\frac{1}{\delta_{If}}\right)e^{-r_{2}(t-t^{*})}+\frac{1}{\delta_{If}}, \ \ t>t^{*},\\ \end{array}\right.  $$

where *δ*_*I*0_ is the diagnosis rate at the initial time. If the efficiency of detection is increasing with time *t*, then the diagnosis rate *δ*_*I*_(*t*) will increase. The parameter *r*_2_ measures how fast the diagnosis rate increases (i.e. the duration of diagnosis decreases) as more medical equipments or resources become available. The final diagnosis rate *δ*_*If*_ is usually larger than *δ*_*I*0_. However, if the medical resource is inadequate, the diagnosis rate *δ*_*I*_(*t*) can decrease and the final diagnosis rate *δ*_*If*_ can be less than *δ*_*I*0_.

According to the basic reproductive number *R*_0_, time-varying contact rate Eq. (2) and diagnosis rate Eq. (3), the effective reproductive number *R*_*c*_(*t*) of time-dependent model is given by the following formula:

$$R_{c}(t)=\frac{\beta\rho c(t)(1-q)}{\delta_{I}(t)+\alpha+\gamma_{I}}+\frac{\beta(1-\rho) c\theta(1-q)}{\gamma_{A}}. $$

Although the government’s mandatory intervention plays a major role in epidemic control, people’s behavior changes such as keeping social distancing, wearing facial masks and washing hands due to media and expert suggestions cannot be ignored. Hence, the piecewise function similar to the previous contact rate and diagnosis rate is applied to the transmission rate *β*. Considering that the impact of behavior change on the spread of the disease is not as great as the government mandatory intervention, the exponential change form is not used. If the number of reported confirmed cases increases, the public will enhance self-protection measures. Thus, we assume that the transmission rate *β* is inversely proportional to reported confirmed cases *H*(*t*). The time-dependent transmission rate *β*(*t*) takes the following form:

4 $$ \beta(t)=\left\{\begin{array}{l} \beta_{0}, \ \ if\ \frac{1}{klog(H(t))}>1,\\ \beta_{0}\frac{1}{klog(H(t))} \\ \end{array}\right.  $$

where *k* represents the indicator measures strength of people’s awareness of self-prevention. The larger the value of *k*, the smaller the transmission rate.

#### Data fitting with autonomous model

**Fig. 2:**
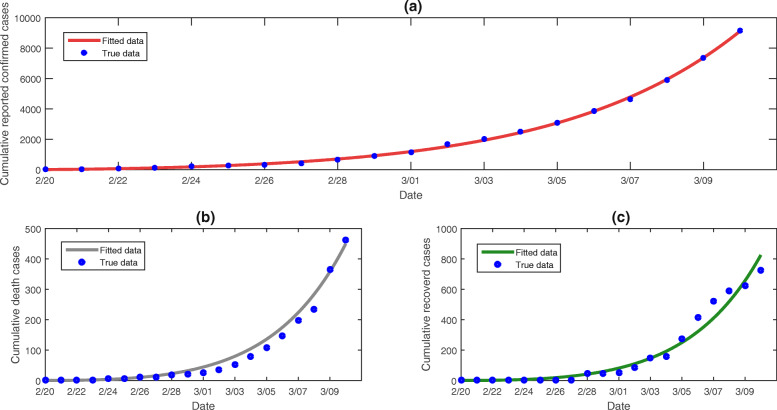
Fitting of the autonomous model to the data of COVID-19 in Italy from February 20 to March 10. (**a**) shows the number of cumulative confirmed cases, (**b**) shows the number of death cases, and (**c**) shows the number of recovered cases

According to the total population of Italy and the epidemic situation on February 20, 2020, we set initial values to be *S*(0)=60 480 000, *H*(0)=3 and *R*(0)=0. According to the WHO [33], the incubation period of COVID-19 is about 7 days. Thus, *σ*=1/7. The quarantined individuals were quarantined for 14 days, thus *λ*=1/14. We obtain other unknown parameter values by fitting data on reported number of cumulative confirmed cases, death cases and recovery cases from February 20 to March 10 in Italy. We utilized the nonlinear least-square (NLES) method in Matlab to fit model solution to the real data sets, as shown in Fig. 2. The estimated parameter values are listed in Table 1.

## Results and discussion

### Global epidemics

**Fig. 3:**
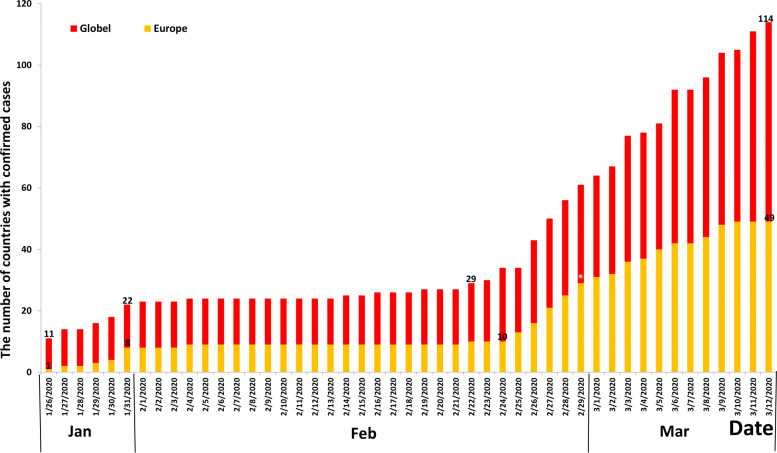
The epidemics in Europe and the world. The red bar represents the number of countries with confirmed cases in the world. The yellow bar represents the number of countries with confirmed cases in Europe

The global spread of the epidemic has gone through two stages, as shown in Fig. 3. In the first stage, COVID-19 spread to 22 countries around the world before the end of January. In this stage, there was a huge deficiency of cognition about this new coronavirus. In early February, under severe travel restrictions of a few cities including Wuhan in China and strict quarantine interventions carried out by other provinces, the epidemic did not spread swiftly to more countries and only increased by seven countries in 30 days window period. Nevertheless, in the second phase, it took only 19 days for the number of infected countries to ascend rapidly from 29 on February 22 to 114 in late March. The discrepancy between these two stages is due to the interventions implemented by China and other countries.

### Data comparison in Italy and Guangdong

According to the World Bank Organization, Italy has 60.48 million people while Guangdong has a population of 111.69 million. Thus, there are more susceptible people in Guangdong than in Italy [34]. However, it can be seen from the data of accumulative cases that it took 19 days for Guangdong Province to increase from 1 case to more than 1000 cases, while it took only 11 days for Italy to increase from 4 cases to more than 1000 cases. It illustrates that the prevention and control tactics of Guangdong Province in the early stage of the epidemic is more effective than that of Italy under the condition of similar diagnosis rate.

**Fig. 4:**
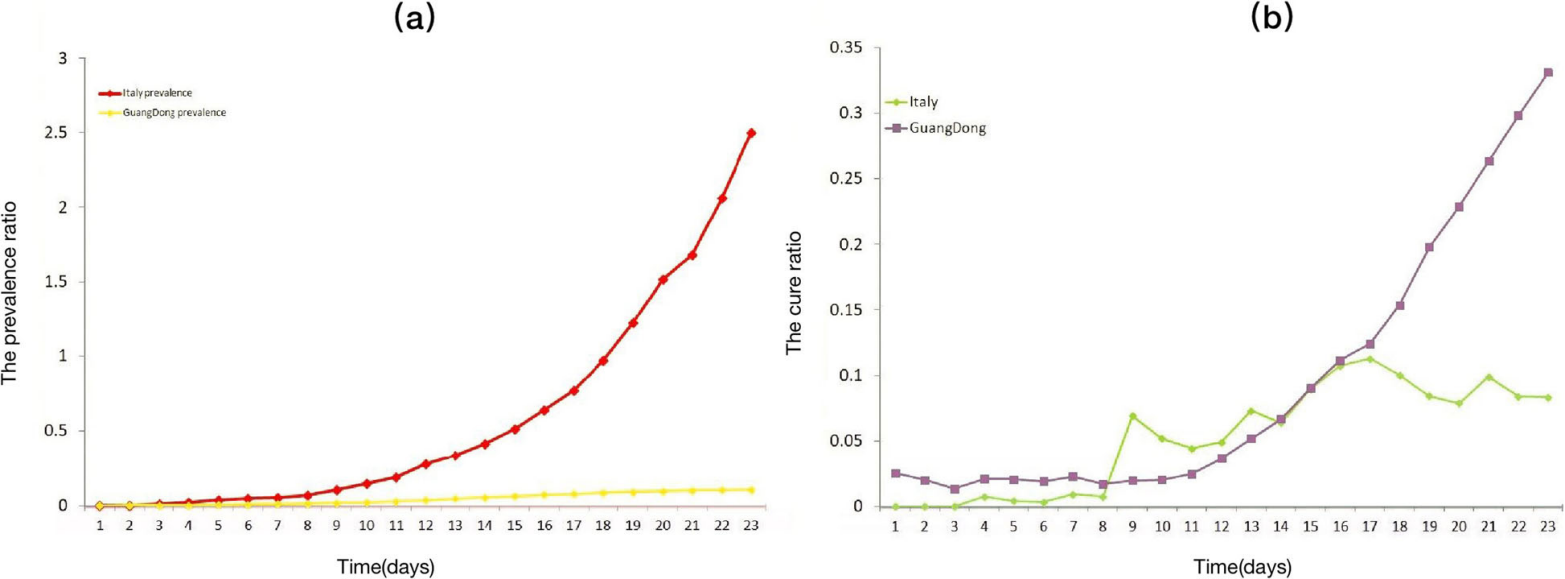
The prevalence ratio (**a**) and cure ratio (**b**) in Italy and Guangdong. The x-coordinate represents time (days) during the period from Feb 20 to Mar 13 in Italy and Jan 19 to Feb 10 in Guangdong

The ascent and descent of newly confirmed cases are bound up with the prevalence ratio, cure ratio and prevention and control measures in the two places. When other conditions are the same, the higher the prevalence ratio, the higher the cure ratio. The more effective the interventions, the fewer newly diagnosed patients per day. Specifically, in Fig. 4 we utilize the ratio of reported cases to the total population to represent the prevalence ratio. Because the calculated prevalence ratios are very small, we multiply by 10 000 for the ease of illustration. Similarly, we use the ratio of recovered cases to cumulative confirmed population to represent the cure ratio. In sum, we select February 20 as Italy’s 1st day and February 19 as Guangdong’s 1st day, with the x-axis representing days and y-axis representing the prevalence ratio or cure ratio. Italian prevalence ratio is much higher than Guangdong’s (Fig. 4a). Italy has more than 20 times as many patients as Guangdong, while Guangdong has nearly twice as many susceptible populations as Italy. This means that Italian epidemic state is more serious than Guangdong’s. In view of the cure ratio (Fig. 4b), we can see that Guangdong’s curve is more smooth than Italy, which means that Guangdong’s cure ratio is more stable than Italy’s.

The data observation and comparison suggest that the situation of the international epidemic, particularly in Italy, was urgent. Compared with the epidemic situation in Guangdong Province, the situation in Italy is relatively serious due to the lack of timely control measures. Last but not least, the cure ratio curve (Fig. 4b) shows that medical resources would be insufficient in Italy.

### Parameter estimation

In the early stage of the outbreak in Italy, the probability of successful transmission was 13.34% (*β*=0.1334). There were 40% asymptomatic infected individuals in infected population and their ability to infect others was 10% of those with symptoms (*ρ*=0.6,*θ*=0.1). The disease-induced death rate among symptomatic was 1.3% (*α*=0.013). Our parameter values are consistent with results in some other publications: the successful infection rate is 12% in reference [35], the ratio of asymptomatic infections is 56% in reference [36], and the case-lethal rate among symptomatic is 1.29% in Italy and 1.3% in New York in references [37, 38]. The initial contact rate *c* was 15 in Italy and 10 in Guangdong, China [8]. This also verified our data comparison analysis that the prevention and control measures implemented in Italy were not sufficiently timely and effective comparing with Guangdong.

According to the estimated values of parameters and the formula of the basic reproductive number, the values of *R*_0_ is 4.3211. It’s close to the result of another publication (*R*_0_=4.10 in Italy) [40]. The basic reproduction number of COVID-19 is higher than that for influenza (1.4 −1.6) [39] and in comparison with the values of SARS epidemics (*R*_0_=4.91) in Beijing in 2003 [41] and MERS (*R*_0_=3.5−6.7) in Jeddah in 2014 [42].

### Uncertainty and sensitive analyses

To examine the possible impact of enhanced interventions on COVID-19 epidemic in Italy, using parameter estimates we plotted the predicted current number of confirmed cases with varying parameters related to the time-dependent contact rate *c*(*t*) and diagnosis rate *δ*_*I*_(*t*) including *τ*, *c*_*b*_, *r*_1_, *δ*_*If*_, *r*_2_ in the model with time-dependent parameters.

The effect of time lag *τ* in the contact rate *c*(*t*) on the epidemic of COVID-19 is shown in Table 1. We can see that the estimated peak value of the number of current confirmed cases significantly increases and the peak time delays 0 - *τ* days as time lag increases. Earlier and stricter lockdown implementation leads to earlier peak time and much lower peak value. For instance, under the control condition *c*_*b*_=4,*r*_1_=0.03,*δ*_*If*_=0.5,*r*_2_=0.05, the number of maximum infected cases is 2.42×10^5^ when *τ*=5. When *τ*=10, the peak value is 4.00×10^5^, which means that confirmed cases increase by 65% when the time lag increases by 5 days. This suggests that the earlier and stricter the blockade, the better the control effect.

**Fig. 5:**
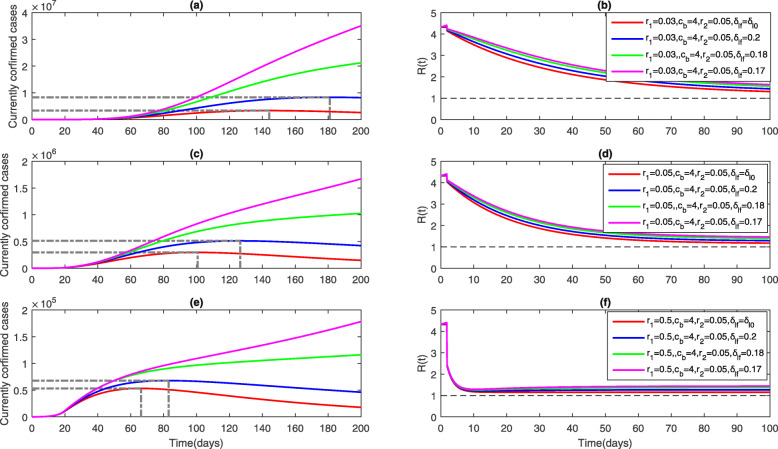
The prediction of currently confirmed cases (left column) and the effective reproduction number (right column) with different sets of parameter values (see text). The x axis is time (days) of simulation. (**a**-**b**) *r*_1_=0.03, (**c**-**d**) *r*_1_=0.05, (**e**-**f**) *r*_1_=0.5. The red, blue, green and magenta lines represent *δ*_*If*_=*δ*_*I*0_, *δ*_*If*_=0.2, *δ*_*If*_=0.18, *δ*_*If*_=0.17, respectively

**Fig. 6:**
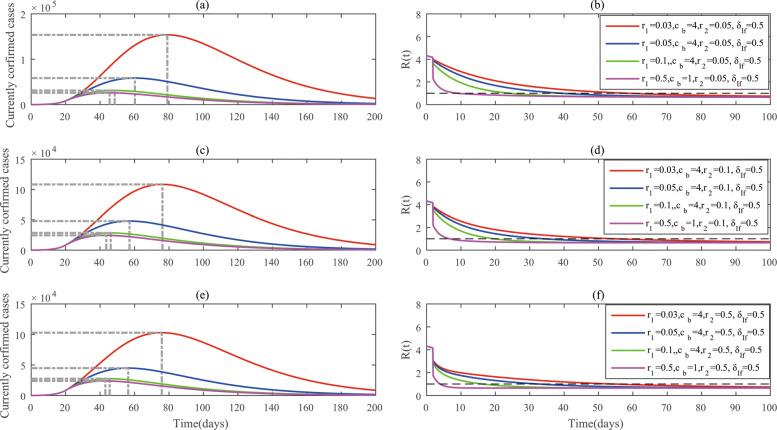
The prediction of currently confirmed cases (left column) and the effective reproduction number (right column) with different sets of parameter values (see text). The x axis is time (days) of simulation. (**a**-**b**) *r*_2_=0.05; (**c**-**d**) *r*_2_=0.1, (**e**-**f**) *r*_2_=0.5. The red, blue, green and magenta line represent *r*_1_=0.03,*c*_*b*_=4, *r*_1_=0.05,*c*_*b*_=4, *r*_1_=0.1,*c*_*b*_=4, *r*_1_=0.5,*c*_*b*_=1, respectively

Figures 5 and 6 show changes of the disease dynamics when the diagnosis rate *δ*_*I*_(*t*) is an increasing function and a decreasing function, respectively. There are in total 72 sets of parameter values, representing the variation in the intensity of the control measures implemented.

Figures of the same color in Fig. 5a, c and e show that improving the efficiency of infection detection can largely affect the spread of the infection. The simulations with four different colors in Fig. 5a, c and e show that when the exponential decreasing rate in the contact rate increases, the number of confirmed cases will decrease and the epidemic will peak earlier. For example, under the control conditions *c*_*b*_=4,*δ*_*If*_=0.5,*r*_2_=0.05,*τ*=0, the estimated accumulated peak value of confirmed cases decreases by 93% (3.36×10^6^−2.96×10^5^) when the exponential decreasing rate *r*_1_ increases by 67% (0.03 −0.05). In addition, the effective reproduction number *R*(*t*) will decrease eventually to less than 1 as the control intensities increase, see Fig. 5b, d and f. Therefore, the stronger the control intensity, the faster the infection goes extinct.

During the outbreak of COVID-19, the number of infections rises, leading to the shortage of medical resources. The detection rate may be smaller. We use *δ*_*If*_≤*δ*_*I*0_ to describe this situation in which *δ*_*I*_(*t*) decreases. The magenta and green curves in Fig. 6a, c and e show that the maximum level of the infection will increase when the minimum diagnosis rate is 0.18 or 0.17. If the minimum diagnosis rate is 0.2 or the diagnosis rate does not increase, the blue and red curves in Fig. 6a, c and e show that the number of confirmed cases is much higher than that in Fig. 5 because of shortage in medical resources. The detailed predicted results of the time when the infection reaches the peak and the maximum number of confirmed cases under different parameter sets are shown in Table 2. These simulations suggest that the effects of prevention and control strategies on epidemic variation are important and huge.

**Table 2:** Different control measure intensities and prediction results

	Parameter assumption	Peak day	Maximum confirmed cases
*c*_*b*_=4, *r*_1_=0.03	*δ*_*If*_=0.5, *r*_2_=0.05, *τ*=0	79	1.54×10^5^
	*δ*_*If*_=0.5, *r*_2_=0.05, *τ*=5	79	2.42×10^5^
	*δ*_*If*_=0.5, *r*_2_=0.05, *τ*=10	80	4.00×10^5^
	*δ*_*If*_=0.5*r*_2_=0.1*τ*=0	76	1.08×10^5^
	*δ*_*If*_=0.5*r*_2_=0.1*τ*=5	77	1.86×10^5^
	*δ*_*If*_=0.5*r*_2_=0.1*τ*=10	77	3.32×10^5^
	*δ*_*If*_=0.5*r*_2_=0.5*τ*=0	76	1.03×10^4^
	*δ*_*If*_=0.5*r*_2_=0.5*τ*=5	76	1.75×10^5^
	*δ*_*If*_=0.5*r*_2_=0.5*τ*=10	77	3.21×10^5^
	*δ*_*If*_=*δ*_*I*0_*r*_2_=0.05*τ*=0	144	3.36×10^6^
	*δ*_*If*_=*δ*_*I*0_*r*_2_=0.05*τ*=5	144	5.38×10^6^
	*δ*_*If*_=*δ*_*I*0_*r*_2_=0.05*τ*=10	144	9.31×10^6^
	*δ*_*If*_=0.2*r*_2_=0.05*τ*=0	181	8.34×10^6^
	*δ*_*If*_=0.2*r*_2_=0.05*τ*=5	181	1.29×10^7^
	*δ*_*If*_=0.2*r*_2_=0.05*τ*=10	181	2.51×10^7^
*c*_*b*_=4, *r*_1_=0.05	*δ*_*If*_=0.5, *r*_2_=0.05*τ*=0	60	5.85×10^4^
	*δ*_*If*_=0.5, *r*_2_=0.05*τ*=5	61	1.05×10^5^
	*δ*_*If*_=0.5, *r*_2_=0.05*τ*=10	63	1.99×10^5^
	*δ*_*If*_=0.5*r*_2_=0.1*τ*=0	57	4.81×10^4^
	*δ*_*If*_=0.5*r*_2_=0.1*τ*=5	59	4.81×10^4^
	*δ*_*If*_=0.5*r*_2_=0.1*τ*=10	61	8.79×10^4^
	*δ*_*If*_=0.5*r*_2_=0.5*τ*=0	56	1.81×10^5^
	*δ*_*If*_=*δ*_*I*0_*r*_2_=0.05*τ*=0	101	2.96×10^5^
	*δ*_*If*_=*δ*_*I*0_*r*_2_=0.05*τ*=5	101	5.77×10^5^
	*δ*_*If*_=*δ*_*I*0_*r*_2_=0.05*τ*=10	101	1.21×10^6^
	*δ*_*If*_=0.2*r*_2_=0.05*τ*=0	127	5.11×10^5^
	*δ*_*If*_=0.2*r*_2_=0.05*τ*=5	127	9.82×10^5^
	*δ*_*If*_=0.2*r*_2_=0.05*τ*=10	127	2.07×10^6^
*c*_*b*_=4, *r*_1_=0.1	*δ*_*If*_=0.5, *r*_2_=0.05*τ*=0	48	3.12×10^4^
	*δ*_*If*_=0.5, *r*_2_=0.05*τ*=5	52	6.69×10^4^
	*δ*_*If*_=0.5, *r*_2_=0.05*τ*=10	55	1.41×10^5^
	*δ*_*If*_=0.5*r*_2_=0.1*τ*=0	46	2.85×10^4^
	*δ*_*If*_=0.5*r*_2_=0.1*τ*=5	50	6.26×10^4^
	*δ*_*If*_=0.5*r*_2_=0.1*τ*=10	54	1.35×10^5^
	*δ*_*If*_=0.5*r*_2_=0.5*τ*=0	46	2.75×10^4^
	*δ*_*If*_=0.5*r*_2_=0.5*τ*=5	49	6.15×10^4^
	*δ*_*If*_=0.5*r*_2_=0.5*τ*=10	54	1.33×10^5^
	*δ*_*If*_=*δ*_*I*0_*r*_2_=0.05*τ*=0	66	5.32×10^4^
	*δ*_*If*_=*δ*_*I*0_*r*_2_=0.05*τ*=5	71	1.56×10^5^
	*δ*_*If*_=*δ*_*I*0_*r*_2_=0.05*τ*=10	76	4.64×10^5^
	*δ*_*If*_=0.2*r*_2_=0.05*τ*=0	83	6.78×10^4^
	*δ*_*If*_=0.2*r*_2_=0.05*τ*=5	88	2.07×10^5^
	*δ*_*If*_=0.2*r*_2_=0.05*τ*=10	89	6.10×10^5^
*c*_*b*_=1, *r*_1_=0.5	*δ*_*If*_=0.5, *r*_2_=0.05*τ*=0	46	2.61×10^4^
	*δ*_*If*_=0.5, *r*_2_=0.05*τ*=5	50	6.00×10^4^
	*δ*_*If*_=0.5, *r*_2_=0.05*τ*=10	54	1.34×10^5^
	*δ*_*If*_=0.5*r*_2_=0.1*τ*=0	44	2.45×10^4^
	*δ*_*If*_=0.5*r*_2_=0.1*τ*=5	48	5.69×10^4^
	*δ*_*If*_=0.5*r*_2_=0.1*τ*=10	53	1.28×10^5^
	*δ*_*If*_=0.5*r*_2_=0.5*τ*=0	43	2.39×10^4^
	*δ*_*If*_=0.5*r*_2_=0.5*τ*=5	48	5.60×10^4^
	*δ*_*If*_=0.5*r*_2_=0.5*τ*=10	53	1.27×10^5^

**Fig. 7:**
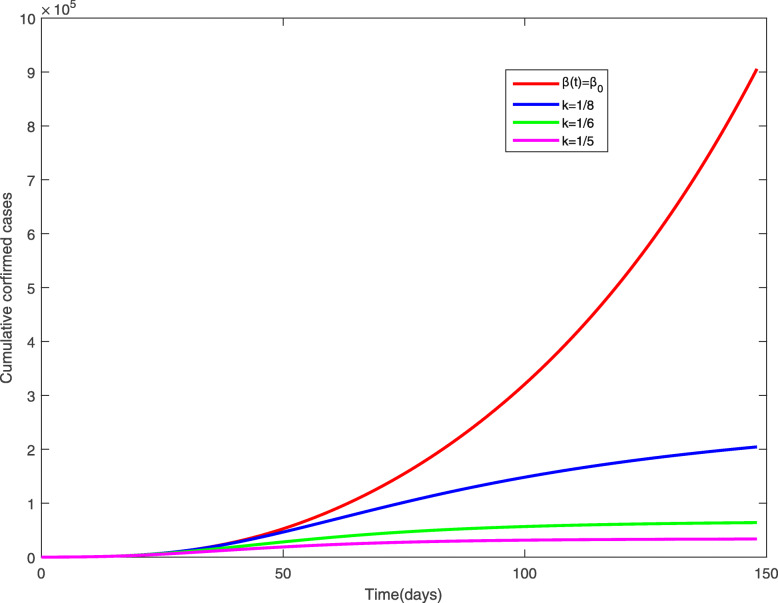
The effect of the public self-protection on the COVID-19 transmission in Italy. The x axis is time (days) of simulation. The red, blue, green and magenta lines represent *β*(*t*)=*β*_0_, *k*=1/8, *k*=1/6, *k*=1/5, respectively

As shown in Fig. 7, we evaluate population’s response to the outbreak at four different levels by setting *k*=1/8,1/6,1/5 in Eq. (4) and *β*(*t*)=*β*_0_. The values of other parameters are the same as those in Table 1. The red curve shows that the cumulative confirmed cases will keep rising and increasing the parameter *k* clearly reduce the number of cumulative confirmed cases and can be effective in slowing the overall disease progression trend. This indicates that media news coverage on the status of disease could help improve people’s self-protection awareness, leading to a better control of the disease outbreak.

**Fig. 8:**
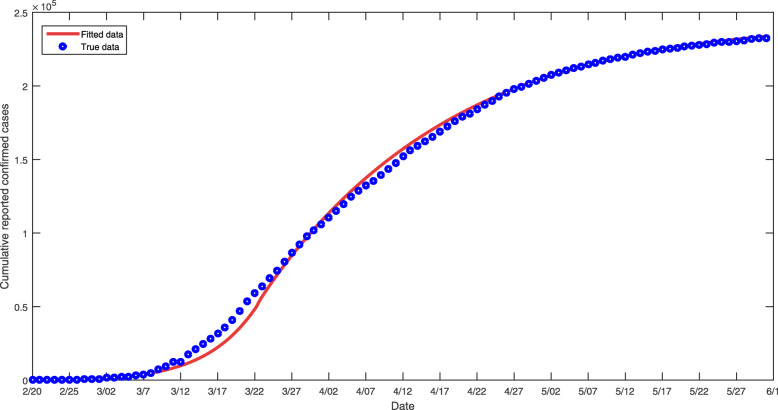
Fitting of the time-dependent model to very recent data on the cumulative confirmed cases from February 31 to May 31. The blue points are data and the red line is the fitting result

Under different parameter settings, we made predictions of the peak time and cases based on the data from February 20 to March 10. The data in later time show that on April 20th the number of current confirmed cases in Italy peaked at 108 237. This is close to one of our predictions: the number of current confirmed cases reaches the peak value 127 000 and the peak time is 53 days (i.e., April 26). Furthermore, using the parameters values in Table 2, we estimated the parameters in the time-dependent model and fit the cumulative reported cases of Italy by the NLES method (Fig. 8). We obtain the parameter estimates *c*_*b*_=2.252,*r*_1_=0.0242,*δ*_*If*_=0.2257,*r*_2_=0.0189,*τ*=12.35. The estimation results show that the decrease in the contact rate is very large, suggesting that Italian recent control measures have played a great role in disease control. The value of the minimum contact rate indicates the strictness of social distancing control and the value of the diagnosis rate determines whether medical resources are sufficient.

## Conclusions

Italy had a very serious epidemic situation before the national lockdown (i.e., March 10), with the basic reproduction number was 4.32. The control measures at that time, including the blockade of schools and medical support, were not sufficiently timely and effectively. Non-pharmaceutical compulsory interventions including contact restrictions (e.g., blockade and quarantine) and improvement of case recognition (i.e., diagnosis rate) play an important role in containing the COVID-19 epidemic. Besides, the effect of individuals behavior change (e.g., keeping social distancing and wearing masks) due to media coverage of the dynamic situation of the epidemic cannot be ignored.

It follows from the data observation and analyses that Italy closed schools at least five days later than Guangdong and the prevalence ratio in Italy is more than 20 times as Guangdong although Italy has fewer susceptible people. The cure ratio curve reveals that there is also a shortage of medical equipments and resources in Italy. Besides, from the global epidemics situation analysis China’s timely lockdown of Wuhan city had provided about 30-day window period, during which (from January 24 to February 22) there were only 7 newly infected countries.

The key parameters including delayed effect of prevention actions *τ*, the exponential decreasing rate of contact rate *r*_1_, the final diagnosis rate *δ*_*If*_, the individual behaviour change constant *k* could significantly affect the outbreak of epidemic COVID-19. In particular, when the estimated peak value of infected cases was controlled at around 108 350 (i.e., *c*_*b*_=4,*r*_1_=0.03,*δ*_*If*_=0.5,*r*_2_=0.1,*τ*=0), it increased by 72% (108 350 −186 420) if the delayed effect of prevention actions *τ* increases by 5 days; or decreased by 56% (108 350 −48 068) if the exponential decreasing rate of contact rate *r*_1_ increased to 0.05; or decreased by 39% (108 350 −66 080) if the final diagnosis rate *δ*_*If*_ increased by 16%.

This study presents a novel methodology through using data analysis and embedding the piecewise continuous function of contact rate, diagnosis rate and individual behavior changes (media reports impact) into the autonomous SEIR-type model, showed that combining data analysis with a mathematical model are beneficial for describing the dynamics of the epidemic from the early stage and quantifying the specific effect of non-pharmaceutical measures including lockdown, medical support and media reports on COVID-19. It demonstrated that these control measures affect the accumulated number of hospital notifications by reducing the contact rate (increasing the exponential decreasing rate of contact rate) and the transmission rate (increasing the individual behaviour change constant), and also increasing the diagnosis rate. All these results confirmed the crucial role of governments in implementing early and strict blockade measures and in reducing detection time, and the importance of the media publicity to improve the public awareness of self-protection. Therefore, for mitigating COVID-19 epidemic, it is recommended that enforcing isolation and blockade orders, strengthen the supplement of medical resources, and improving the public self-protection awareness should be implemented timely and effectively.

## Data Availability

Not applicable.
